# Impact and Persistence of *Serratia marcescens* in *Tenebrio molitor* Larvae and Feed under Optimal and Stressed Mass Rearing Conditions

**DOI:** 10.3390/insects13050458

**Published:** 2022-05-12

**Authors:** Florent Dupriez, Agnès Rejasse, Alfredo Rios, Thomas Lefebvre, Christina Nielsen-LeRoux

**Affiliations:** 1 Ynsect, 1 Rue Pierre Fontaine, 91000 Evry, France; florent.dupriez@ynsect.com (F.D.); alfredo.rios@ynsect.com (A.R.); thomas.lefebvre@ynsect.com (T.L.); 2 Micalis Institute, INRAE, AgroParisTech, Université Paris-Saclay, 78350 Jouy-en-Josas, France; agnes.rejasse@inrae.fr

**Keywords:** food safety, yellow mealworm, *Serratia* *marcescens*, microbial persistence, qPCR, insect for food and feed

## Abstract

**Simple Summary:**

A few insects are actually considered for mass production as an alternative protein source for animal feed, notably in the fish and poultry sector. Industrial insect rearing, aims at producing high quality insects, following secure sanitary conditions. As for other livestock’s optimal rearing conditions should avoid stress and pathogens. In this study we investigated how abiotic stresses (sieving, starvation and density) and the presence of the bacteria, *Serratia marcescens,* an opportunistic human and insect pathogen, affect the growth and survival of the yellow mealworm, *Tenebrio molitor*. We also set up tests to determine if this bacterium could survive in the rearing system by analysing over time its persistence in the feed, in the insect and in the feces. Our result indicate that the studied *Serratia* strain is not very virulent to the yellow mealworm, that it can persist in the rearing system and can be detected easily by using a selective growth medium. The stress factors showed no impact from sieving but positive importance was found from high larval density while starvation should be avoided. *Serratia* could be considered as a potential marker, in the assessment of infectious pressure in a rearing system, and should be monitored for sanitary risk issues as well.

**Abstract:**

Industrial insect mass rearing aims to produce quality insects under safe sanitary conditions which can be compromised by pathogens and abiotic stressors. Therefore, knowledge on pathogen persistence, virulence and means of detection is of importance. This study focuses on the opportunistic pathogen *Serratia marcescens* (*Sm*) as a possible candidate to reveal sanitary issues in *Tenebrio molitor* (*Tm*) breeding. A screening test was performed to assess the impact of abiotic stressors (starvation, density and sieving) in presence and absence of *Sm*. Two *Sm* detection methods were conducted, and the kinetics of *Sm* persistence were investigated. Our results show that (i) the presence of *Sm* had a low but significant effect on *Tm* mortality, (ii) a short temporary starvation period had a negative impact on larval growth, (iii) the detection of *Sm* by q-PCR was sensitive but less convenient than a specific *Sm* growth media, (iv) the kinetics of persistence showed that *Sm* declined but survived for nine days in the feed and in the feces for three weeks. Both the relatively low virulence and the persistence in the environment suggest that *Sm* could be used as an indicator for the sanitary status of mealworm production.

## 1. Introduction

Mass production of insects for human and animal consumption could help counter global food insecurity, organic waste disposal and promote economic and social development [1]. The insect sector is now entering a crucial stage of industrialization with several large-scale projects of fully automated vertical farms expected to produce several thousands of tons of insects per year (IPIFF) https://ipiff.org accessed on 15 March 2022. The yellow mealworm *Tenebrio molitor* is one of the species with potential for large scale production, intended to be transformed into protein for animal feed as well as for human food following its authorization by EFSA [2]. Such an intensification is new for insect farming, and many challenges are faced, among which the prevention of infection and diseases are the biggest. Indeed, industrial insect farming’s first goal is to produce high-quality insects, in a short time, following safety and secure sanitary conditions. The sustainability of this new industry by using by-products or waste as raw material has shown a potential positive environmental impact [3].

However, the impact of pathogens on insect health might be enhanced by abiotic stress factors due to industrialization, which can increase the potential contamination rate and persistence of pathogens in insect livestock [4,5].

Several abiotic stress factors can be considered for insect mass rearing and the impact and persistence of pathogenic bacteria. The high flow of materials in vertical farms requires the use of large equipment and robots to carry out rearing operations. The machines’ accuracy level or technical issues can lead to three main stresses on insects: (1) a lack of feed, resulting in starvation and intraspecific competition; (2) over-densification of larval/adult populations in rearing crates, which could impact mealworms’ environment and performances [5,6] and (3) sieving/separation mechanical stress, which might damage them. These stress factors challenge mealworms’ health by reducing their defense and immunity or by changing their environment and behavior. In such context, pathogen prevalence could increase and create sanitary risk for the livestock as for the final insect product [7]. Many insect pathogens, belonging to various groups such as viruses, bacteria, fungi, protists and nematodes, can infect insects produced for food and feed, as described by Eilenberg et al. (2015) [8]. Although few diseases have been referenced so far in mealworm production, the emergence of new pathogenic issues must be considered as it is a common feature for every livestock industry. In addition, it is an issue to consider the possible presence and persistence of opportunistic human pathogens in the insect production system [9] for sanitary risk evaluation.

Among the possible microbial sources of pathogens, the most common one is the gut microbiota of the insect itself, sometimes contaminated by the feed, sometimes inherited and considered as commensals. Several studies have shown a large variation in the bacterial microbiota associated with *Tm* [10,11,12], of which some potential pathogenic species have been detected. Otherwise, with respect to sanitary issues, food-borne and environmental pathogens like *Listeria monocytogenes*, *Staphylococcus aureus*, *Escherichia coli* and *Clostridium perfringens* have so far not been recorded as being present in the gut microbiome of *Tm* [10,11]. Meanwhile, two studies have reported on the ability of *Salmonella enterica* to persist in *Tm* gut and feces, following artificial contamination of the feed substrate [7,13]. In these studies, it was found that *Salmonella* could persist in the insect, largely dependent on the contamination load, but there was no clear mention of pathogen development in the insect.

Apart from insect pathogens, whether specialists or generalists, which have the ability to overcome the insect’s defense (such as *Bacillus thuringiensis* or *Hypocreales* and *Entomophtorales* fungi), microbes that take advantage of the weakening of the host by a stress (or primary infection) are called opportunistic pathogens. As such, they are good indicators of the health status of livestock subjected to various stressors. In this study, we focus on *Serratia marcescens* (*Sm*), an opportunistic Gram-negative pathogen that can be found in a wide range of environments like soil, water and plant surfaces. Contaminated larvae can easily be detected due to pink/red coloring as a typical (but not systematic) symptom. *Sm* strains have even been studied for their potential as biocontrol agents [14]. Furthermore, *Sm* is also an opportunistic pathogen of animals and humans, especially involved in hospital-acquired infections (HAIs) in immune-depressive patients [15]. In several publications, the pathogenicity of *Sm* has been studied in the context of insect microbiota and diseases. The source of infection seems to be more attributed to the development of bacteria already present in the insect gut, in connection with the degradation of rearing conditions, than to the pathogen spread from individual to individual. Indeed, the virulence level depends on the insect species, the insect stage of development, and the stress status [16]. In the case of minor presence in the gut, *Sm* is not pathogenic [17]. In contrast, if *Sm* reaches the insect hemocoel, the immune system is quickly overwhelmed, and the host dies from sepsis [18]. Recently in honeybees [19] it was reported that *Sm* may be virulent under some conditions. *Sm* was also isolated from *Tm* in a screening search for proteolytic bacteria [20], indicating that *Sm* is sometimes naturally associated with mealworms. Similarly, in next-generation sequencing, the genera *Serratia* was found to be associated with the microbiota of *Tm* [10,11,20]. In a challenge immune priming assay in *Tm*, *Sm* and other pathogens remained virulent [21].

Therefore, in this study, we aimed to explore whether the above-mentioned abiotic stresses, i.e., larval densification, starvation and mechanical disturbance by sieving could increase the impact of *Sm*. We also evaluated the *Sm* persistence in *Tm* larvae, feces and in the feeding substrate to assess the potential spread of *Sm* in the rearing system as an opportunistic pathogen. We tested two methods for determining the bacterial load of this pathogen, one based on specific quantitative PCR and the other based on growth on a selective medium.

## 2. Materials and Methods

### 2.1. Tenebrio Molitor Rearing and Diet

*Tm* larvae from Ynsect’s R&D insectarium (Evry, France) were used for the experiments. Prior to their use, the larvae were kept in the feeding substrate grinded wheat bran (Soufflet, Moulins de Corbeil, France) which was supplemented with hydrocolloid water gel as supplied by the company, in plastic trays (39.5 × 34 × 19.1 cm). The larvae were reared at 26 °C and 60% of relative humidity (RH).

### 2.2. Strain and Culture

*Serratia marcescens (Sm)* strain Bizio 1823, DSM n°3012 (isolated from pond water), was purchased from Deutsche Sammlung von Mikroorganismen und Zellkulturen (DSMZ). This strain is considered an environmental strain. The strain was cultured in LB (Luria-Bertani) or BHI (Brain Heart Infusion) media at 30 °C with shaking at 200 rpm, for the majority of the detection and infection experiments. The generation time in these growing conditions was 30 min. In addition, a (*Sm*) selective medium with 0.5% erythritol [22] was used for the analysis focusing on *Sm* persistence in *Tm* feces. Colony quantification was performed with serial dilution of the various samples on LB, BHI or erythritol agar plates using glass beads for spread.

### 2.3. Abiotic Stress Factors Combined with Sm Infection

**Figure 1:**
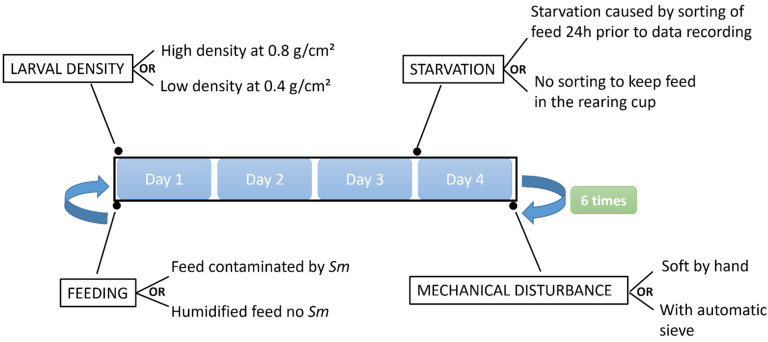
Experimental design. The figure shows the time schedule of experimental setup: when the feed was delivered, the data collections and when stresses were applied. The experiment lasted for 24 days.

Three abiotic stressors were tested: starvation, larval densification and sieving. Starvation consisted in taking away the feed substrate present in the tray 24 h prior to data collection as shown in Figure 1: Two larval densities were tested, a high density (0.8 g/cm^2^ of larvae; approximatively 2000 larvae per cup), and a low density (0.4 g/cm^2^ of larvae; approximatively 1000 larvae per cup) with a mean larval weight of 14 mg, about 6 weeks after egg hatching. Sieving was applied to separate the larvae from the feces. High sieving stress was applied for 30 s by the automatic sieve SYSMO from 3R Company, while low sieving stress was applied by separating the larvae by manual sieving using sieves with a diameter of 200 mm and a mesh of 0.8 mm from Retsch Company, Eragny, France).

To assess the influence of *Sm*, 0.3 mL/g feed of a 24 h culture (30 °C, 200 rpm, BHI medium) was added. The culture optical density was measured by spectrophotometry at 420 nm and was used at an optical density around 9, corresponding to the stationary growth phase and a concentration of 6 × 10^10^ CFU/mL. This experimental design is summarized in Figure 1 and Table 1.

### 2.4. Experimental Design

Squared plastic cups with a surface of 36 cm^2^ were used to house the larvae. To screen for the relevant factors and their interactions, a nonreplicated 2-level full-factorial experiment was set up. This screening design was chosen as the objective was to screen for a large number of potential interactions given that there were four factors being studied [20]. Cups of larvae calibrated at 14 mg were used and provided with humid (30%) wheat bran feed. For starved larvae, feed was moved away either by soft manual sieving or by automatic sieving, the day before data taking, while for the non-starved larvae, feed was added into the cup as soon as it became limited. 

### 2.5. Variables Measured

During the experiment, the following variables were measured:

Different larval Key Performance Indicators, KPI’s, were mesured to evaluate larval performance. Among these, the relative individual mean mass gain wax estimated by dividing the difference of the final and initial Indiviudal Mean Mass (IMM) over the initial IMM. Here, the IMM was estimated using the mean weight of 100 larvae. Similarly, the relative larval mass gain of the rearing container during the experiment was calculated by substracting the final and initial container larval mass and dividing it by the initial larval mass. Larval mortality was computed by dividing the difference in the number of larvae at the start and end of the trial over the initial number of larvae. 

The number of larvae was computed by dividing the larval mass in the tray over the individual mean mass. Finally, Feed Conversion Ratio (FCR) was estimated by dividing the feed mass consumed at the end of the rearing period over the tray larval mass gain. All the measurements were done in fresh weight.

$$IMM=\frac{Mass\;of\;100\;larvae}{number\;of\;larvae}$$

$$IMM\;relative\;gain=\frac{IMM_{END}-IMM_{INITIAL}}{IMM_{INITIAL}}$$

$$Larval\;Mass\;relative\;gain=\frac{Larval\;Mass_{END}-Larval\;Mass_{INITIAL}}{Larval\;Mass_{INITIAL}}$$

$$Larvae\;number=\frac{Larval\;Mass}{IMM}$$

$$Mortality=\frac{Larvae\;number_{INITIAL}-Larvae\;number_{END}}{Larvae\;number_{INITIAL}}$$

$$FCR=\frac{Total\;Feed\;Consumed}{Total\;larval\;mass\;gained}$$

### 2.6. Statistical Analyses: Stress Factors

In order to screen for the interactions between the stress factors and *Sm* virulence, half normal plots were used. This is a graphical procedure used to separate nonzero regression coefficients for nonreplicated designs. These coefficients in two-level designs correspond to the difference between the two levels averages. The function LGB from the statistical software R package was used to generate the half normal plots. Given that factors do not have an impact on the response, this collection of differences should resemble to a normally distributed sample with zero mean [23]. The half normal scores plotted against the absolute value of estimated effects were used to identify parameter estimates that were unlikely to occur due to random variation. Analyses of variance (ANOVA) were then used to assess the significance of the main effects and the interactions that were identified by the half normal plots. If the ANOVA’s indicated a significant effect, Tukey post hoc tests (package emmeans in R) were used to determine if there were differences among the different factors’ levels. Similarly, given that group variances where unequal, a group variance structure was fitted using a generalized least-squares regression model in the R-package nlme. 

### 2.7. In Vivo Experiment for Quantification of Sm by qPCR in Larva and Feces

To define the detection threshold of *Sm* in *Tm*, a specific and sensitive qPCR approach was evaluated based on a larval feeding experiment. The test was set up using batches each consisting of five larvae placed in small glass Petri dishes. The presence of *Sm* was searched in (i) whole larvae, (three batches), (ii) in dissected whole intestines (three other batches) and from (iii) feces recovered from the 3 batches. Before the feeding assay, all the larvae were starved for 24 h. Then, 5 larvae (12 mg each) were fed during 16 h on 20 mg of wheat bran mixed with 25 µL of a 24 h *Sm* culture OD_420_ = 9 or with LB medium alone (control). The larvae consumed approximately 50% of the feed. Before processing, the larvae were cleaned with ethanol 70% *w*/*w* and dried for 2 min. Whole larvae were directly frozen in nitrogen and stored at −80 °C before DNA extraction. The intestine samples were obtained by cutting the head and gently pulling out the digestive tube (food bolus and gut) with fine tweezers from larvae placed on ice and were directly frozen in liquid nitrogen and stored at −80 °C. To study the presence of *Sm* in the feces, the larvae were cleaned and placed in clean small glass Petri dishes for 24 h before collecting feces and recording their weight. The feces were then also frozen in liquid nitrogen and stored at −80 °C before DNA extraction.

DNA extraction was performed following the recommendations from the supplier of a PowerLyser PowerSoil DNA Isolation Kit (MO BIO laboratories, Inc. Ozyme, St. Quentin en Yvelines, France). A lysis step was added by cell disruption for 2 × 40 s at 4 m/s using FastPrep-24™ Classic from MP Biomedicals. The concentration of DNA was measured with Nanodrop 2000 (Thermo Scientific, Illkirch, France). qPCR was performed with a PCR Max qPCR Kit for *Sm* (company Cole Parmer ref PKIT 10139, Villepinte, France). The PCR Max qPCR Kit for *Sm* also has an internal extraction control. A control DNA is added when extracting DNA after the FastPrep ^®^ treatment from the sample and is detected through the VIC channel during qPCR. VIC is a positive control for the DNA extraction process. The positive *Sm* control generates the *Sm* standard curve (number of copy of the porin gene (*ompC*)/Ct value).

The qPCR standard curve is necessary to perform the quantitative analysis. Six successive dilutions to 1/10 were carried out, then, 5 μL of each dilution was analyzed by qPCR. The number of Ct’s was plotted on the graph, knowing the number of copies of the positive control, the standard curve was created, and the number of copies of an unknown sample could be plotted. The kit also contained an endogenous control: a poor endogenous control signal may indicate that the sample did not contain sufficient biological material. The data were first analyzed with QuantStudio™ 12K Flex Software v1.2.2, and further analyzes were performed using the Thermo Fisher ConnectTM cloud.

### 2.8. Persistence of Sm in Inoculated Wheat Bran Feed

An alternative *Sm* detection method was sought using an erythritol-based selective substrate for the growth of *Sm* [22]. This medium contained 0.5% erythritol as the sole carbon source and allowed the growth of only *Sm* and principally no other bacteria. *Sm* overnight cultures at 6 × 10^10^ CFU/mL (OD_420_ = 9) were used to soak the wheat bran; 6 × 10^9^ CFU (100 µL) was mixed with 80 mg of wheat bran. Twenty-one replicates in Petri dishes were placed in an incubator at 30 °C. Each following day (7 days for a period of 9 days), three replicates were analyzed by mixing the 80 mg bran with 500 µL of physiological water. One hundred microliters of the suspension were spread on erythritol medium plate, and bacteria outgrowth (CFU) was counted after 2 days at 30 °C.

### 2.9. Monitoring of Sm Persistence in Tm Feces

The presence of *Sm* in the feces or wheat bran was evaluated after homogenization with a pestle in 250 µL of sterile NaCl 0.9%, and *Sm* quantification was performed with serial dilution and spreading on BHI and erythritol agar plates. The persistence of *Sm* in *Tm* feces was based on free feeding on *Sm*-contaminated wheat bran. The assays (Figure 7) were run with 4 batches of 5 larvae (50 mg each). All batches were first placed overnight without feed to empty the gut before the assay. Next, each batch was fed ad libitum on 80 mg wheat bran mixed with 100 µL of overnight culture in BHI at 30 °C OD_420_ = 9 for 24 h. The feed was in excess, and the larvae ate only about the half of the given feed. The larvae were cleaned after each step with ethanol 70%. Between each feces sampling, the larvae were moved to small glass Petri dishes, as it was easier to collect the feces from glass (no static electricity). Twenty-four hours after the start of larvae feeding, the feces were sampled during 2 h (24–26 h). Another sampling was performed again from time 30 h to 32 h. The third sample was taken from feces recovered at 48 h (excretion from 32 h to 48 h). Then, the larvae were kept in clean glass Petri dishes for two hours to be sure that no more feces were excreted. Next, the larvae were fed ad libitum with wheat bran without *Sm* for 6 h with the aim of evaluating whether *Sm* persisted in the gut cleared for feed/feces from the first feeding. Feces were again collected after 16 h (point 56–72 h) and on day 4 and day 19. The amount of feces collected per batch/larva varied from point to point (depending on time from feed separation). CFU counting was based on 2 mg of feces, which corresponded to about 30 feces particles.

## 3. Results

### 3.1. Impact of Stressors on Rearing Performances

**Figure 2:**
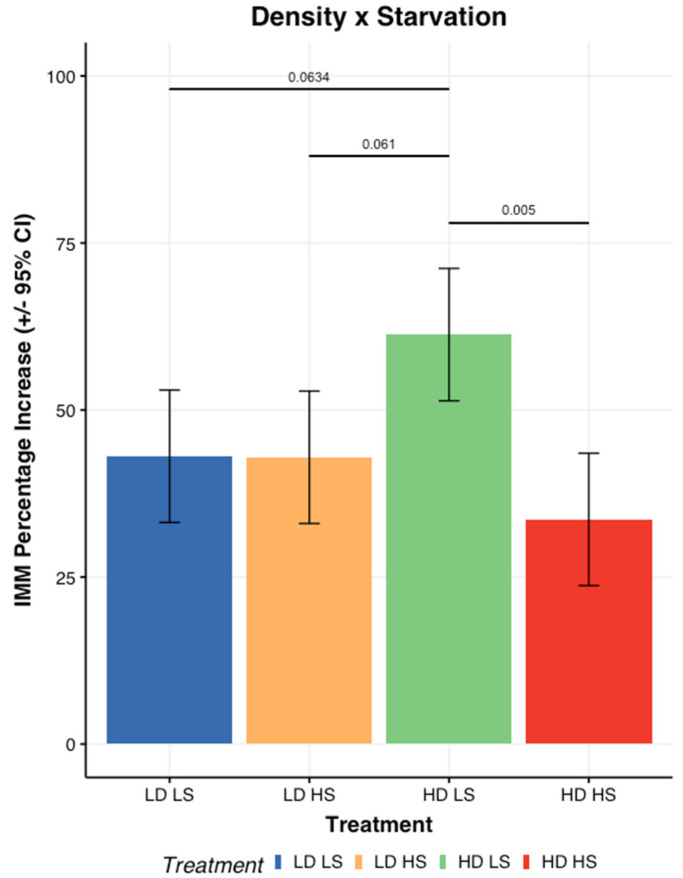
Impact of starvation and density on Individual Mean Mass increase. The IMM relative gain is expressed as a percentage. In this figure, LD and HD stand for low and high larval density, while LS and HS stand for no-starvation and starvation, respectively.

Among the different abiotic stress factors tested, density and starvation seemed to have the most impact on physiological factors. A half normal plot for IMM relative gain indicated an interaction between density and starvation. A subsequent analysis of variance (ANOVA) confirmed a significant interaction between density and starvation level (F_1,12_ = 9.15, *p* = 0.011). Figure 2 shows a statistically significant difference between starved larvae and non-starved larvae at high density. The IMM relative gain for the high-density non-starved larvae was approximately 61.3% and 33.6% for the starved larvae. No significant differences could be detected between the IMM relative gain at low density between the larvae that were starved (42.6%) or not (43.1%). Finally, no significant differences were detected among the high-density starved larvae and the low-density ones.

- *Larval mass relative growth and Feed Conversion Ratio (FCR)*

**Figure 3:**
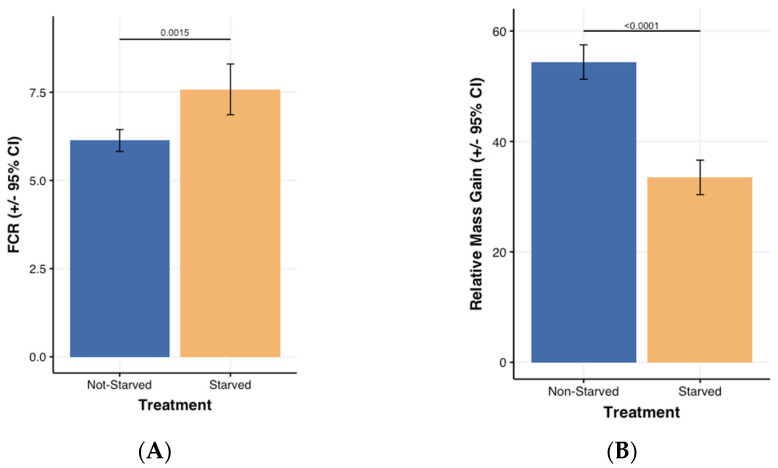
(**A**,**B**) respectively, represent FCR (Feed Conversion Ratio) and the relative mass gain expressed as a percentage and as a function of starvation after 24 days.

The half normal plot identified starvation as the single factor affecting relative larval mass gain. Figure 3A shows that larvae mass relative gain (F_1,14_ = 103, *p* < 0.0001) as expected was higher (54%) in the non-starved group compared to that in the starved one (33.5%.) Similarly, half normal plots indicated that starvation impacted FCR. The starved larvae had higher FCR than the non-starved ones did (6.13 vs. 7.58; t = −4.38 and *p* = 0.0015; (Figure 3B).

- *Stress impact on mortality*

**Figure 4:**
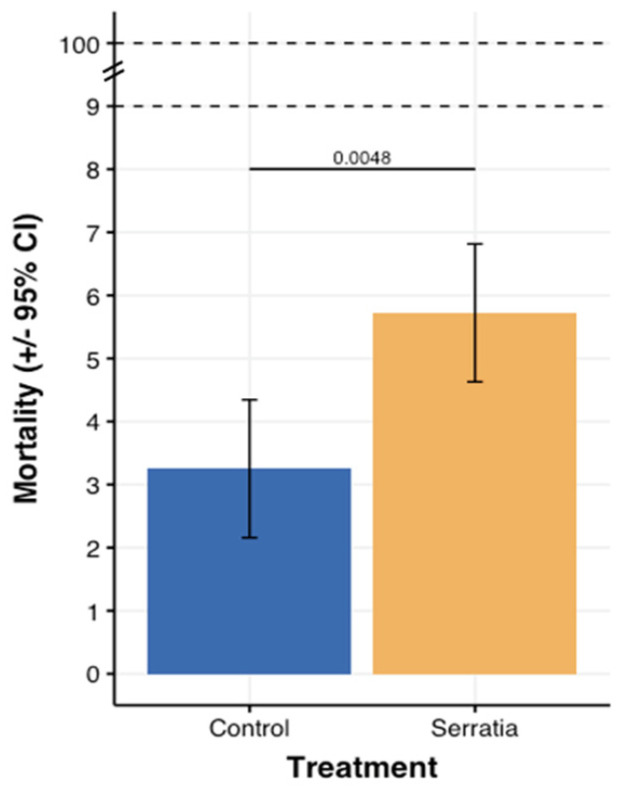
Larval mortality after 24 days, “Control” corresponds to the cups without the addition of *Serratia* to the feed while “Serratia” indicates the cups in which the bacterial culture was added to the feed at the beginning of the experiment.

Among the parameters evaluated in this study, *Sm* feed inoculation was the only factor identified to have an impact on mortality upon half normal plot examination. Residuals showed some patterns left in ANOVA that only included this factor. To improve the fit of the model, the interaction Density × Sieving (the second most important term) and its lower order terms were added. None of these terms were significant. *Sm*-inoculated feed (5.72%) induced a slightly higher larval mortality rate (F1,11 = 12.399, *p* < 0.005; Figure 4) than in those given non-inoculated feed (3.25%). 

### 3.2. Detection of Sm by qPCR

**Figure 5:**
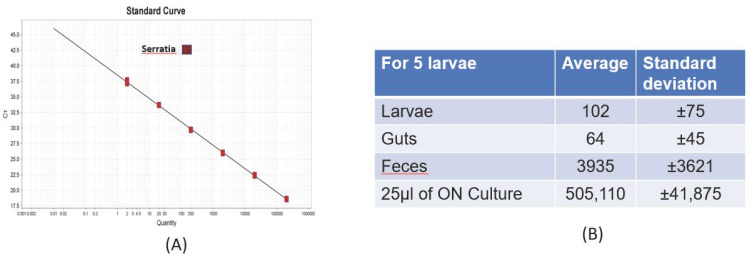
(**A**) The qPCR standard curve, necessary to perform the quantitative analysis, was made with the positive control supplied in the kit. The number of Ct was plotted on the graph. Knowing the number of copies of the positive control, the standard curve was created, and the number of copies of an unknown sample could be plotted. The lower the Ct, the higher the number of bacteria. (**B**) The numbers refer to the quantity of *Sm* bacteria extrapolated from qPCR analysis on DNA extracted from different *Tm* samples and the quantity of *Sm* in an overnight culture used to inoculate wheat bran consumed by *Tm*.

In this study, we aimed at testing methods which could detect the presence of *Sm* at low density in *Tm* larvae, in the feeding substrate and the feces, to evaluate the pathogen persistence and the potential risk for rearing. First, we targeted the use of a commercial kit. A PCR Max qPCR Kit for *Sm* detected as few as two copies of the *Sm*-specific porin (*ompC*) gene, indicating high sensitivity at least in pure *Sm* cultures (Figure 5). The kit contained a positive *Sm* control to generate the *Sm* standard curve (number of copies of the porin gene (*ompC*)/Ct value). This positive control template showed that the primers and the probes for detecting the targets *Sm* gene worked properly.

Indeed, qPCR Ct values were 28 ± 3 in the control, in a pure culture, in feces (about 5 mg) and in feed (20 mg), indicating that the extraction efficiency was good for such sample types. However, the detection capacity for *Sm* in whole *Tm* larva and the gut samples were very low (Figure 5B); indeed here, the Ct values were higher than 31, which indicated that the extraction process was not optimal for *Tm* tissue.

#### Monitoring of *Sm* Persistence in Wheat Bran and Feces on Erythritol Selective Medium

**Figure 6:**
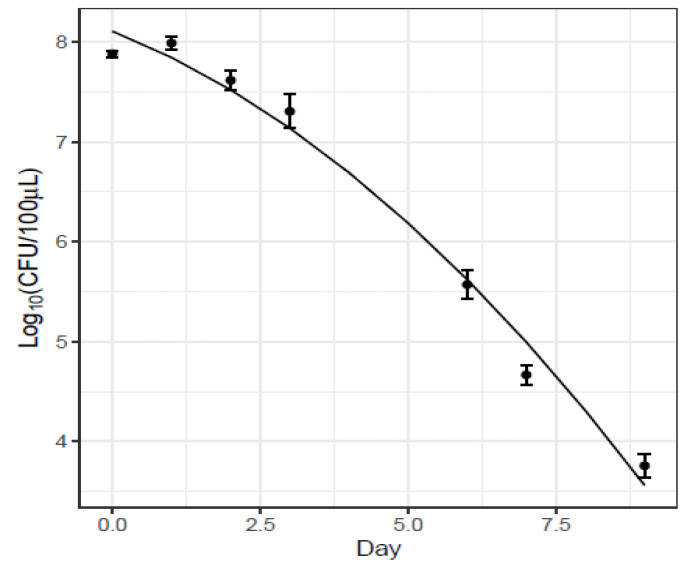
Quantity of *Serratia* detected (on erythritol plates) during 9 days from 80 mg bran inoculated with 6 × 10^9^ CFU *Sm* culture and stored at 30 °C. Mean of 3 batches SEM (standard error of the mean).

In order to get a better estimation for the capacity of *Sm* to persist in *Tm* rearing conditions, we investigated another and simpler method, the use of a *Sm*-selective growth medium with erythritol. Prior to the experiment, we investigated the possible natural presence of *Sm* in *Tm* and in wheat bran on the selective erythritol growth medium and also on the global bacterial outgrowth on the nonspecific growth medium BHI. No *Sm* was found on the erythritol medium, but on BHI, about 10^3^ non-identified bacterial colonies were found and between 3 × 10^2^ and 1 × 10^4^ colonies were recorded in the feces from each larva (collected during 2 h), while in 50 mg of wheat bran feed, only about 50 colonies were found. Next, we investigated the capacity of *Sm* to survive in the contaminated feed under rearing conditions. The experiment showed that the persistence of *Sm* on wheat bran at 30 °C slowly decreased with time, going from 10^8^ at the start of the assay to about 10^4^ CFU in 80 mg bran after nine days (Figure 6, Table 2).

**Figure 7:**
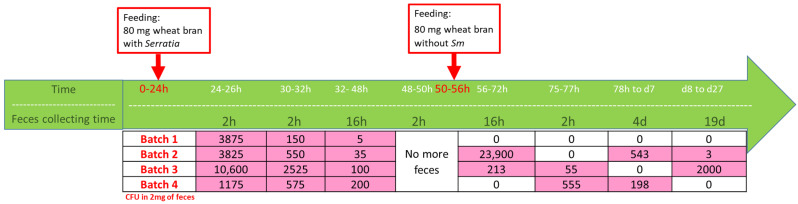
The kinetics of the presence of *Serratia marcescens (Sm*) in *Tm* feces. The numbers are *Sm* CFU’s (on erythritol medium) from 2 mg of feces, according to time after consumption (0–24 h) of *Sm*-contaminated wheat bran. The experiment lasted for 27 days but feces were collected from day 2 (24–48 h) with a certain interval until no more feces were excreted (48–50 h) and again after consumption of feed without *Sm* during hours 50–56 h. Although no feed was added, feces and *Sm* were still collected from day 8 to day 27 (19 d) h (hours), d (days).

To follow the kinetics of the persistence of *Sm* in the *Tm*, larvae were fed with *Sm*-inoculated feed. The protocol used is illustrated in Figure 7. Pathogen persistence was measured in the feces, based on the hypothesis that if *Sm* was still present in the feces, following a period where larvae were provided non-inoculated wheat bran, it should indicate that *Sm* could remain for a certain time in *Tm’s* digestive tube and therefore inform about its persistence. The results are indicated in Figure 7 as the number of colonies growing on the erythritol plates from a suspension of feces. The data showed that there was a certain level of variation among batches, each containing five larvae. *Sm* could disappear after two days or to the opposite, survive and sometimes multiply after three days. For instance, in batch 4, *Sm* appeared in the feces after three days while it was not detected in the 56–72 h period. Perhaps *Sm* could bind to the gut and be excreted later when the larvae were fed again as observed in batch 2 and batch 3. It was not possible to separate the feces from the larvae immediately after excretion and it was probably that *Tm* ate feces and therefore could be contaminated again with *Sm*. This might explain the differences among batches, and therefore a precise (number) estimation of persistence in the larvae proved difficult. However, the data indicate the possibility that *Sm* can survive and multiply in a rearing system at least for a period of about three weeks. 

## 4. Discussion

The production of insects at an industrial scale presents a set of challenges, including insect production losses due to pathogenic agents. Studies investigating the interaction of diverse environmental/mechanical stresses and pathogens are typically conducted at a laboratory scale where insects are exposed to stress conditions that might differ substantially from those found in industrial mass rearing. A typical example is the study of larval density impact. Academic studies use low larval densities (lower than 0.05 g/cm^2^), while industrial production densities are set higher than 0.5 g/cm^2^. Similarly, mechanical stress can be underestimated due to differences in sieve size/type and sieving frequencies. The purpose of this study was to assess the impact of industrial practices/environment and their interaction with the opportunistic *Sm* pathogen on larval mass gain and insect health as well as the pathogen’s persistence kinetics during *Tm* rearing. 

In order to screen for several potential interactions between the stress factors and *Sm* and their impact on the virulence of *Sm* on *Tm*, we used a one-replicate multifactorial design. From this design and analysises we could only observe a small absolute impact of *Sm* on larval mortality, but we could not detect any interactions of *Sm* with the other stress factors. Specifically, this study showed a slight increase in mortality due to *Sm*, from 3.25 to 5.72%. In typical *Tm* industrial production, mortality rates vary from 3 to 4%, due to competition, genetics or environmental conditions. We expect that this mortality rate variation should have only a small consequential impact on rearing performance. This does not mean that such interactions were not present, just suggests that the pathogenicity of *Sm* is not enhanced by the factors tested to detectable levels as it is an opportunistic pathogen. Nevertheless, if they exist, we expect them to be of small biological significance. Conversely, the stresses applied might not have been severe enough to lower the larval immunity [24]. Moreover, our experiment was set up with 14 mg larva, which are less sensitive to pathogens than younger instars are [25]. Therefore, in industrial rearing, the presence of *Sm* could have a higher impact if present on the feed just after egg hatching. In regard to IMM relative gain, we found a statistically significant difference between starved larvae and non-starved larvae at high larval densities. These results indicate that at the same high density (0.8 g/cm^2^), if there is a lack of feed for one day, even if it is compensated the day after, the temporarily starved larvae will not be able to individually grow as much as larvae with continuous feeding. Interestingly, the one-day starvation, did not affect the IMM relative gain at low larval densities. One explanation might be that *Tm* larvae grow faster at high temperature, which can be created when the population is denser, resulting in increased metabolism and better FCR by reaching an optimal temperature [26]. Similarly, as expected, we found lower larval mass relative gain and higher FCR for the starved larvae.

It is important to avoid pathogens in industrial insect rearing, and therefore detection of and information on how a pathogen persists in the rearing environment, the feed, the insect and the feces are needed. In this study, we tested two detection methods, one based on specific qPCR and one by plating on a selective growth medium. For the qPCR approach, our results indicated that the tested DNA extraction method was not effective for larvae and guts, although it has shown to be efficient for *Galleria mellonella* larvae (not published). Therefore, a better adapted tissue preparation method is needed for *Tm*, which has higher content of chitin. However, the qPCR method was precise and could be used for detection of *Sm* at low prevalence in wheat bran and feces. In addition, this method (Taqman primers and the specific kit) is expensive and needs technical expertise. The second method was based on the outgrowth of *Sm* on a selective growth medium. This method was applied to the persistence experiment and permitted to record the kinetics of *Sm* survival in wheat bran and feces. This method is easy to use and permits not only identifying *Sm* but also knowing if *Sm* is alive, which is not the case for PCR-based detection. To increase the detection probability at low numbers of *Sm* in routine production monitoring, amplification though culturing in selective medium before plating could be done. In several metagenomic analyses targeting the gut microbiota from various organisms (mice, humans, etc.), feces are considered as a relevant indicator for presence or absence of bacteria or other microorganisms. In this study, we only searched to recover *Sm* from the feces as an indicator for its persistence and survival in *Tm*. The results showed that *Sm* could persist a certain time, but we found strong variation among replicates indicating that for the studied *Sm* strain, no strong amplification was observed. Meanwhile, our study indicates that the long-term survival was higher in the larvae/feces than in the wheat bran alone. Analyses of the dynamics and survival of human pathogens during entomoconversion of various substrates are reported for *Tm* and especially for *Hermetia illucens* [27]. *Tm* studies on the persistence of *Salmonella* indicated that the survival was clearly dose-dependent, and no multiplication was observed [7,13]. Our objective was to evaluate the capacity of a non-*Tm* adapted *Sm* strain to contaminate and persist in *Tm* mass rearing, therefore, we used an environmental strain with no information on virulence. Whether other *Sm* strains, as reported in [18], or other entomopathogens like *B. thuringiensis serovar tenebrionis* [28] with known pathogenicity for *Tm*, could persist better in similar conditions, is of importance for the risk evaluation of the *Tm* production system and for the downstream products (insect meal and frass).

## 5. Conclusions

Industrialization of an animal production system raises the question of the disturbance of the livestock’s living conditions and consequently of the animals’ welfare. In the case of insects, and more specifically mealworms, it is challenging to estimate the impact of stress factors induced by abiotic and mechanical operations. In this study, we were not able to detect an interaction of the stressors (density, starvation and sieving) with the *Sm* strain used. Nevertheless, the observed mortality was slightly higher for the larvae fed with *Sm*-inoculated wheat bran. This seems to indicate that at the stressor levels tested for the specific time/life-stage period, these stressors had little or no impact on enhancing the pathogenicity of *Sm* on *Tm* given our experimental conditions. Similarly, as expected, an impact on growth and FCR was observed when larvae were exposed to small periods of starvation. Meanwhile, in case of a more virulent *Sm* strain, detection of *Sm* in *Tm* rearing might be critical. Indeed, this study revealed the capacity of *Sm* to persist into the feeding substrate and in the insect even at a low level. This low prevalence opportunistic bacterium, known to infect physiologically weakened insects, while being harmless at normal conditions, could therefore be an appropriate indicator to assess livestock sanitary status. Detection using an erythritol-based selective medium appeared to be an effective and rapid monitoring method. Molecular techniques of detection by qPCR were less appropriate, but would require further investigation, particularly in the DNA extraction step. The monitoring of the sanitary state of an insect farm is indeed a field still explored little, but it is a real challenge for the development of feed and food insect industry. The identification of markers or microbial indicators, as they exist in other animal husbandries, would be a major asset.

## Figures and Tables

**Table 1:** Experimental design description for each of the 16 treatment combinations.

One Cup/Treatment	Larval Density	Sieving	Starvation	Serratia
**1**	0.4 g/cm^2^	Manual	No	Water
**2**	0.4 g/cm^2^	Manual	Yes	Water
**3**	0.4 g/cm^2^	Mechanical	Yes	Water
**4**	0.4 g/cm^2^	Mechanical	Yes	Inoculum
**5**	0.4 g/cm^2^	Manual	No	Inoculum
**6**	0.4 g/cm^2^	Mechanical	No	Water
**7**	0.4 g/cm^2^	Manual	Yes	Inoculum
**8**	0.4 g/cm^2^	Mechanical	No	Inoculum
**9**	0.8 g/cm^2^	Manual	No	Water
**10**	0.8 g/cm^2^	Mechanical	No	Water
**11**	0.8 g/cm^2^	Mechanical	Yes	Water
**12**	0.8 g/cm^2^	Manual	Yes	Water
**13**	0.8 g/cm^2^	Mechanical	No	Inoculum
**14**	0.8 g/cm^2^	Mechanical	Yes	Inoculum
**15**	0.8 g/cm^2^	Manual	No	Inoculum
**16**	0.8 g/cm^2^	Manual	Yes	Inoculum

**Table 2:** Regression parameter estimates for the Quantity of *Serratia* detected during 9 days from 80 mg bran inoculated with 6 × 10^9^ CFU *Sm* culture and stored at 30 °C.

Parameter	Value	Standard Error	*p*-Value
Intercept	8.11	0.12	7.43 × 10^−23^
Day	−0.23	0.07	5.9 × 10^−3^
Day^2^	−0.03	8 × 10^−3^	1.5 × 10^−3^

## Data Availability

The data presented in this study are available in article.
